# Multi-Omics Provides New Insights into the Aroma Regulation of *Rhododendron fortunei Lindl* Treated with Methyl Jasmonate and Brassinosteroids

**DOI:** 10.3390/cimb47121054

**Published:** 2025-12-16

**Authors:** Danyidie Zhang, Yi Qin, Jiayi Wu, Xingyun Zhong, Haichao Hu, Zhihui Chen, Pei Shi, Yueyan Wu

**Affiliations:** 1 College of Biological and Environmental Sciences, Zhejiang Wanli University, Ningbo 315100, China; 2 College of Life Sciences, Dundee University, Dundee DD1 4HN, UK

**Keywords:** *Rhododendron*, aroma, brassinosteroid (BR), methyl jasmonate (MeJA), multi-omics

## Abstract

*Rhododendron fortunei Lindl* is known for its unique aroma, but the molecular mechanism behind plant hormone-mediated aroma biosynthesis remains unclear. To explore how brassinosteroids (BRs) and methyl jasmonate (MeJA) regulate its aroma, this study analyzed *R. fortunei* petal samples via physiological assays, volatile metabolome analysis, and transcriptome sequencing. Physiologically, BR/MeJA significantly increased the superoxide dismutase (SOD) and catalase (CAT) activity and decreased the malondialdehyde (MDA) content. Metabolome analysis identified 1268 volatile organic compounds (VOCs), with 265/70 VOCs up-/downregulated in the BR group and 248/181 VOCs up-/downregulated in the MeJA group compared to the controls. Transcriptome sequencing identified 19,333 differentially expressed genes (DEGs), which were enriched in pathways such as terpenoid and polyketide metabolism. Multi-omics screening revealed the candidate gene *RfCYP92C6*, whose transient overexpression in *Nicotiana benthamiana* increased the terpenoid content 2.2-fold. These findings clarify the aroma regulation mechanism of BRs/MeJA in *R. fortunei* and support the improvement of its aroma traits via genetic engineering.

## 1. Introduction

The floral scent is one of the core characteristics of ornamental plants [1,2]. During plant pollination processes, the floral scent can play multiple roles, including attracting pollinators and enhancing the pollination efficiency, while also helping plants to resist external stresses [3]. The floral scent is composed of numerous low-molecular-weight, low-boiling-point, and volatile compounds. To date, more than 1700 floral volatile compounds (VOCs) have been identified, including terpenoids, esters, benzenoids, and amino acid derivatives; these compounds act synergistically to form the unique aroma characteristics of plants [4,5]. *Rhododendron fortunei Lindl* belongs to the subgenus Hymenanthes (evergreen rhododendrons). It is mainly distributed in areas at altitudes of 620–2000 m in South China, with large, light pink or light purple flowers, an elegant shape, and a rich aroma. It possesses both high ornamental and economic value [6]. With the rapid development of the economy and society, the demand for high-quality rhododendrons is increasing. Although many scholars have engaged in breeding research on fragrant rhododendron varieties, our current understanding of the biosynthesis and regulatory mechanisms underlying its aroma remains relatively limited. The biosynthesis of floral scent compounds primarily occurs through three key metabolic groups: terpenoids, phenylpropanoids/benzenoids, and fatty acid derivatives [7]. Among them, terpenes are synthesized through two different pathways in plants. The mevalonate (MVA) pathway occurs in the cytoplasm, while the 2-C-methyl-D-erythritol 4-phosphate (MEP) pathway exists in the plastids [8,9].

Studies have shown that plant hormones not only play an important role in plant growth and development but also regulate aroma biosynthesis [10,11,12]. Brassinosteroids (BRs), as steroidal hormones unique to plants, are crucial for plant growth and development [13,14,15]. In *Arabidopsis thaliana* the foliar application of BRs can protect plants from heavy metal stress [16]; in apples, exogenous BR treatment can promote an increase in the number of lateral roots and improvements in other growth traits [17]. Jasmonic acid and its derivatives are collectively referred to as jasmonates (JAs). Among them, methyl jasmonate (MeJA), one of the current research hotspots, is widely involved in plant physiological metabolic processes and growth and development. In *Muscat Hamburg* grapes, MeJA treatment can significantly increase the content of most monoterpenes [18]; in *Oryza sativa* (rice), MeJA can protect plants against oxidative stress and photosynthetic damage caused by mechanical injury [19].

Superoxide dismutase (SOD), catalase (CAT), and peroxidase (POD) are key protective enzymes in plants and the most important reactive oxygen species (ROS) scavenging systems [20,21]. Malondialdehyde (MDA) is a metabolite produced by the peroxidation of membrane lipids in response to ROS. Its content can reflect the degree of plant cell membrane damage and stress resistance [22,23]. Li et al. found that *Ficus parvifolia* can reduce the MDA content by increasing the activity of antioxidant enzymes [24]; Kamran et al. conducted foliar application on *Tanacetum balsamita L* and found that the FeSO_4_ and NaCl salinity levels affect the content of MDA and CAT [25]; in *Triticum aestivum L.*, the exogenous application of spermine or spermidine under artificially simulated high-temperature conditions significantly increased the activity of SOD, POD, and CAT and reduced the MDA content [26]. By detecting these indicators, researchers can effectively evaluate the physiological health statuses of plants [27].

In recent years, with the development of high-throughput omics technologies, the accumulation and formation mechanisms of floral VOCs have been elucidated in a variety of ornamental plants, such as *Rosa laevigata Michx* [28], *Dendrobium loddigesii* [29], *Lonicera japonica* [30], *Jasminum sambac* [31], and *Camellia japonica* [32]. The combined application of multi-omics technologies helps to elucidate the underlying mechanisms from a molecular perspective. However, studies that reveal the compositions and regulatory mechanisms of floral aroma VOCs in *Rhododendron* species through integrated multi-omics analyses remain relatively scarce. To further explore the effects of different plant hormones on floral aromas, this study treated the petals of *R. fortunei* with a BR and MeJA. Through comprehensive omics analyses (metabolic pathway profiling, volatile compound detection, and regulatory gene analysis), we investigated the mechanisms of action of exogenous hormones in *R. fortunei* aroma formation and screened candidate genes sensitive to BR and MeJA treatments to perform preliminary functional verification. This study will help to better reveal the characteristic differences in unique aroma substances in *R. fortunei* and provides new insights and approaches for the study of aromas in other flowers.

## 2. Materials and Methods

### 2.1. Plant Materials and Related Treatments

**Figure 1:**
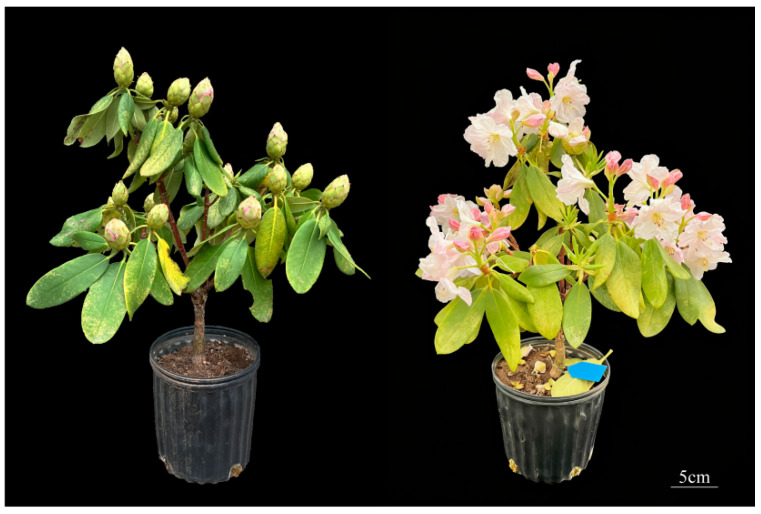
Experimental materials. The left side shows the flower bud stage, which was the period for the first hormone treatment; the right side shows the full blooming stage, which was the period for sample collection. The 5 cm scale bar in the bottom-right corner provides clear visualization of the growth dimensions of the plants.

Experimental *R. fortunei* plants were grown in Siming Mountain National Forest Park (Ningbo City, Zhejiang Province, China) under consistent cultivation and management conditions applied to all plants. Plants with robust growth, no pests or diseases, and uniform vigor were selected (Figure 1). These plants were evenly sprayed with BR (0.2 mg·L^−1^) and MeJA (200 μmol·L^−1^) solutions. (Kangsheng Biotechnology Co., Ltd., Ningbo, China) The control group (designated as CK) was sprayed with clear water containing 0.1% Tween 80 (Solarbio Science & Technology Co., Ltd., Beijing, China); 0.1% Tween 80 was included in all treatment and control solutions to ensure uniform adhesion. Spraying was conducted at the flower bud stage, with a second application at the flower bud swelling stage. The spraying standard was that the plant surface was fully moist without droplet runoff, with a spraying volume of 20 mL per plant. Each biological replicate consisted of 4 plants, with 3 replicates in total. Sampling was performed at the full blooming stage of the plants; during sampling, tissues such as pistils, stamens, and receptacles were removed, retaining only the petals. Among these, petal samples from 3 biological replicates were used for transcriptome analysis and 6 biological replicates for metabolome analysis. In addition, samples of wild-type *R. fortunei* at 4 growth stages (designated as S1–S4) were collected simultaneously, with the stages defined as S1 (flower bud stage), S2 (tightly closed, unswollen flower bud stage), S3 (fully open petal stage), and S4 (early petal senescence stage). Additionally, wild-type samples at the full blooming stage were separated into 5 tissue types: receptacle, stamen, pedicel, pistil, and petal. All aforementioned samples were used for the subsequent analysis of gene spatiotemporal expression patterns. All collected samples were immediately frozen in liquid nitrogen and stored at −80 °C for subsequent analysis.

### 2.2. Determination of Physiological Indicators

First, 0.1 g *R. fortunei* petal samples were accurately weighed, to which 1 mL of phosphate buffer extract (pH 7.4) was added; samples were homogenized on ice and then centrifuged at 12,000 rpm and 4 °C for 10 min. The supernatant was collected and placed on ice for subsequent determination. Petal enzyme activity (SOD, CAT) and MDA content were measured using a commercial kit (Shanghai Future Industry Co., Ltd., Shanghai, China), strictly following the kit’s instructions. All assays included 3 biological replicates.

### 2.3. Determination of VOCs

A sample of 3 g was accurately weighed in a 20 mL headspace vial, 5 mL saturated sodium chloride solution (NaCl) was added, and it was immediately sealed for metabolomic analysis by HS-SPME-GC-MS. The analysis was performed using a TRACE 1610 GC chromatograph equipped with an TriPlus RSH SMART headspace autosampler and coupled to an Orbitrap Exploris mass spectrometry (Thermo Fisher Scientific, Waltham, MA, USA) at Majorbio Bio-Pharm Technology Co., Ltd. (Shanghai, China). Firstly, the volatile compounds were extracted via the HS-SPME technique, employing an SPME Arrow fiber (DVB/Carbon WR/PDMS) (120 μm × 20 mm) with a diameter of 1.1 mm (Thermo Fisher Scientific, Waltham, MA, USA). The HS-SPME conditions were as follows: the incubation and extraction temperatures were 80 °C, the incubation time was 20 min, the extraction time was 10 min, the aging station temperature of the fiber head was 240 °C, the fiber conditioning pre-desorb time was 2 min, the fiber conditioning post-desorb time was 2 min, the desorption time of the sample was 1 min, and the volume of the sample bottle was 20 mL. Then, the fiber was injected into the GC-MS system in split mode for analysis. The injection volume of samples was 1 µL and they were introduced in splitting mode (10:1). The samples were separated with a VF-WAXms (25 m × 0.25 mm × 0.2 µm) capillary column, using 99.999% helium as a carrier gas at a constant flow rate (1 mL/min). The inlet temperature was 240 °C. The GC column temperature was programmed to be held at 40 °C for 0 min and risen to 120 °C at a rate of 8 °C per minute, rising to 230 °C at a rate of 20 °C per minute, and maintained for 4.5 min. The total running time was 20 min.

The mass spectrometer conditions were as follows. The ion source type was the inert electron impact (EI) ionization source. The ionization voltage was 70 eV. The ion source temperature was 250 °C. The scanning mode was full-scan mode, the quality scanning range was *m*/*z* 35–500, and the resolving power was 30,000 full width at half maximum (FWHM). To evaluate the stability of the analytical system during the run-on process, a quality control (QC) sample was prepared during the experiment. QC samples were produced by mixing all test samples and were treated in the same way as formal samples. During instrument testing, three QC samples were dispersedly inserted. The repeatability of QC samples reflected the stability of the instrument in the whole analysis process. At the same time, it could also be used to find variables with large variations in the analysis system to ensure the reliability of the results. The data matrix obtained by searching the database was uploaded to the Majorbio cloud platform (https://cloud.majorbio.com, accessed on 1 July 2025) for data analysis. Firstly, the data matrix was pre-processed as follows. At least 50% of the metabolic features detected in any set of samples were retained. After filtering, for specific samples with metabolite levels below the lower limit of quantification, the minimum metabolite value was estimated and each metabolic signature was normalized to the sum. To reduce errors caused by sample preparation and instrument instability, the response intensities of the samples’ mass spectrometry peaks were normalized using the sum normalization method to obtain the normalized data matrix. Meanwhile, the data were log10 logarithmicized to obtain the final data matrix for subsequent analysis.

### 2.4. Analysis of Differential Metabolites (DAMs)

The R package ropls (Version 1.6.2) was used to perform principal component analysis (PCA) and orthogonal partial least squares discriminant analysis (OPLS-DA) on the pre-processed data matrix, and model stability was assessed via 7-fold cross-validation. The Kyoto Encyclopedia of Genes and Genomes (KEGG) database was used to annotate metabolic pathways for DAMs. Pathway enrichment analysis was conducted using Python’s scipy.stats module (https://docs.scipy.org/doc/scipy/reference/stats.html, accessed on 5 July 2025), and significant biological pathways were identified through Fisher’s exact test (*p* < 0.05).

### 2.5. Determination of Transcriptome Data

Total RNA from *R. fortunei* petals was extracted according to the instructions of the Total RNA Extraction Kit for Polysaccharide- and Polyphenol-Rich Plants (Vazyme Biotech Co., Ltd., Nanjing, China). RNA quality was assessed using agarose gel electrophoresis, a NanoPhotometer Spectrophotometer, a Qubit 2.0 Fluorometer (Thermo Fisher Scientific, Waltham, MA, USA) and an Agilent 2100 Bioanalyzer (Agilent Technologies, Santa Clara, CA, USA) were used to ensure that high-quality RNA was obtained. After library construction and qualification, sequencing was performed on the Illumina sequencing platform. Raw data were filtered to remove invalid data, and high-quality sequencing data were obtained through additional quality control steps, including the analysis of the sequencing error rate distribution and GC content. Fragments per kilobase of transcript per million mapped reads (FPKM) was used to normalize and quantify gene expression levels. The transcriptome data were assembled to generate unigene sequences to obtain sequences, and novel genes were aligned with sequences from the KEGG, GO, NR, Swiss-Prot, TrEMBL, and KOG databases (E-value < 1 × 10^−5^) to complete the annotation of novel genes. Differentially expressed genes (DEGs) were screened based on the fold change and false discovery rate (FDR). DEGs were mapped to each term in the Gene Ontology (GO) database (http://www.geneontology.org/, accessed on 10 June 2025), and the number of DEGs in each term was calculated; pathway enrichment analysis was performed using KEGG pathways as the unit to screen pathways significantly enriched with DEGs, thereby identifying the core biochemical metabolic pathways and signal transduction pathways that DEGs were involved in.

### 2.6. Quantitative Real-Time PCR (qRT-PCR) Validation

Twelve DEGs were randomly selected for validation via qRT-PCR. Total RNA from *R. fortunei* petals (the same samples used for transcriptome sequencing) was reverse-transcribed into complementary DNA (cDNA) using the GoScript™ Reverse Transcription Kit (Promega Corporation, Beijing, China). Following the instructions of the SYBR Green^®^ Premix Ex Taq™ Kit (TaKaRa Bio, Inc., Otsu, Japan), qRT-PCR assays were performed on a Bio-Rad CFX Opus 384 Real-Time Quantitative PCR System (Bio-Rad Laboratories, Hercules, CA, USA). The PCR program was set as follows: pre-denaturation—95 °C for 3 min; amplification cycles (40 total)—95 °C for 10 s (denaturation) at primer-specific annealing temperatures for 30 s and 72 °C for 30 s (extension), with melting curve analysis to confirm the amplification specificity. The relative expression levels of target genes were calculated using the 2^ΔΔCt^ method, using EF-1α as the internal reference gene. Three biological replicates were included for each sample. Primers used for qRT-PCR in this section are listed in Table S1.

### 2.7. Integrated Analysis of Transcriptome and Metabolome

To integrate transcriptome and metabolome data, DAMs and DEGs were subjected to correlation analysis. Pearson correlation coefficients between DEGs and DAMs were calculated to screen for DAM-DEG pairs with the strongest correlations; meanwhile, common DEGs (shared by the BR and MeJA treatment groups) were selected for annotation using the Swiss-Prot database. The absolute value of the correlation coefficient (|r|) reflected the strength of the association between DEGs and DAMs:|r| ≥ 0.7—strong correlation; 0.4 ≤ |r| < 0.7—moderate correlation; 0.2 ≤ |r| < 0.4—weak correlation; |r| < 0.2—very weak or negligible correlation. Correlation significance was assessed using *p*-values, with *p* < 0.05 indicating a significant correlation and *p* < 0.001 indicating an extremely significant correlation.

### 2.8. Cloning and Subcellular Localization of RfCYP92C6

The coding sequence (CDS) of *RfCYP92C6* was obtained from the assembled transcriptome data of *R. fortunei*. First, total RNA was reverse-transcribed into cDNA using the PrimeScript™ RT Kit (with gDNA Eraser, TaKaRa, Dalian, China); then, the *RfCYP92C6* CDS was amplified via PCR using PrimeSTAR^®^ Max DNA Polymerase (TaKaRa, Dalian, China). For the subcellular localization experiment, the *RfCYP92C6* CDS was inserted into the pCAMBIA1300-GFP vector via homologous recombination. After constructing the recombinant vector, it was transformed into *Agrobacterium tumefaciens* str. GV3101 The transformed Agrobacterium was cultured, collected by centrifugation, and resuspended in infiltration buffer (10 mM MES, 10 mM MgCl_2_, 200 μM acetosyringone, pH 5.8) to an OD_600_ of 0.4. The bacterial suspension was aspirated with a syringe and injected into the abaxial surfaces of 4-week-old *Nicotiana benthamiana* leaves, and the injected plants were cultured in a dark growth chamber at 25 °C for 1 day.

### 2.9. Analysis of Spatiotemporal Expression Pattern of RfCYP92C6

Total RNA was extracted from *R. fortunei* samples from different growth stages (S1–S4) and tissue types (receptacle, stamen, pedicel, pistil, petal), following the same protocol as described in Section 2.5. A qRT-PCR was conducted to determine *RfCYP92C6* expression, adopting the method detailed in Section 2.6.

### 2.10. Verification of Gene Transcription Pattern Under Transient Overexpression of RfCYP92C6 in N. benthamiana via qRT-PCR

Transient overexpression experiments were conducted following the method described in Section 2.8, and, after 3 days, infiltrated *N. benthamiana* leaves were harvested: one part was used for qRT-PCR analysis to confirm *RfCYP92C6* transcriptional levels, and the other part was immediately placed into headspace vials for volatile compound detection. For the control group, *Agrobacterium tumefaciens* harboring the empty pCAMBIA1300 vector served as the negative control.

### 2.11. Statistical Analysis

All statistical analyses—including principal component analysis (PCA); hierarchical cluster analysis (HCA); Venn diagram, upset plot, and volcano plot construction; and K-means clustering analysis—were conducted using specific R packages in the R software (R version 4.4.0; https://www.r-project.org, accessed on 28 August 2025). Identified metabolites were annotated using the KEGG Compound Database (https://www.kegg.jp/kegg/compound/, accessed on 28 August 2025). Annotated metabolites were then mapped to the KEGG Pathway Database (https://www.kegg.jp/kegg/pathway.html, accessed on 28 August 2025). Subsequently, pathways containing significantly different metabolites were subjected to metabolite set enrichment analysis (MSEA), with the significance threshold set at *p* ≤ 0.05.

## 3. Results

### 3.1. Effects of Different Hormone Treatments on Physiological and Biochemical Indices of R. fortunei

**Figure 2:**
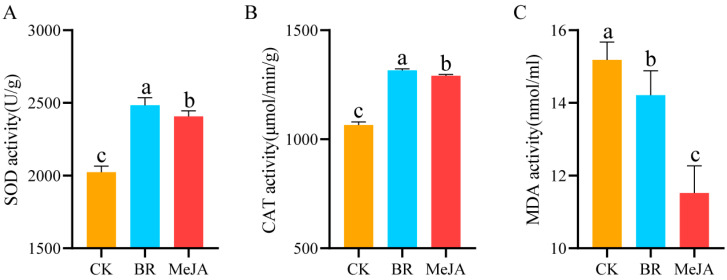
Determination of physiological and biochemical indices. (**A**) SOD activity; (**B**) CAT activity; (**C**) MDA content; Different letters (a, b, c) at the tops of columns indicate significant differences based on multiple comparisons (*p* < 0.05).

The activity of antioxidant enzymes (SOD, CAT) and the MDA content in *R. fortunei* petals under different hormone treatments were quantified, with the results shown in Figure 2. The results indicated that, compared with the control group, the activity of SOD and CAT in the two hormone treatment groups was significantly higher (*p* < 0.05), while the MDA content was significantly lower (*p* < 0.05). Specifically, after the two hormone treatments, SOD activity in *R. fortunei* petals showed the most significant increase, followed by CAT activity—suggesting that SOD is the key antioxidant enzyme in *R. fortunei* petals in response to hormone treatments. Based on these physiological changes, it can be preliminarily inferred that exogenous BRs and MeJA may promote the aroma formation of *R. fortunei* by activating the antioxidant enzyme system.

### 3.2. Analysis of Volatile Metabolome After Different Hormone Treatments

**Figure 3:**
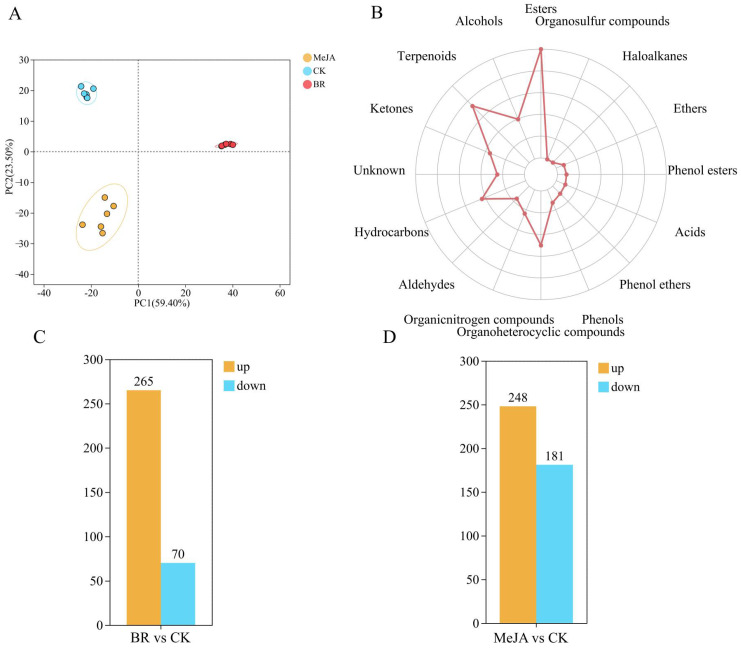
Metabolome analysis of *R fortunei*. (**A**) PCA plot of volatile metabolome. The distance between each coordinate point represents the degree of aggregation and dispersion among samples; the closer the distance, the higher the similarity between samples. (**B**) Radar chart for statistical analysis of VOC categories. The outermost labels represent VOC categories, and the polyline represents the number of metabolites corresponding to each volatile compound category. (**C**) Number of DEGs identified in the BR treatment group. (**D**) Number of DEGs identified in the MeJA treatment group. Orange represents upregulated DEGs, and blue represents downregulated DEGs; the x-axis represents different treatment groups, and the y-axis represents the number of DEGs.

To clarify the overall metabolic differences and variation patterns among groups, PCA was performed to analyze the metabolic characteristics of samples in each group (Figure 3A). The results showed that the two principal components explained 59.4% and 23.5% of the metabolic variation in the three groups of samples, respectively, cumulatively explaining 82.9% of the total metabolic variation; additionally, the BR and MeJA treatment groups were clearly separated from the CK group, indicating significant metabolic differences between the hormone treatment groups and the CK group. This may be associated with the hormone-induced increase or decrease in metabolite content. A total of 16 categories and 1268 types of VOCs were detected in the three groups of *R. fortunei* petal samples (CK, BR, MeJA) (Figure 3B and Table S2). Specifically, these categories included esters (268), terpenoids (199), organoheterocyclic compounds (136), hydrocarbons (119), alcohols (109), ketones (98), unknown compounds (70), organic nitrogen compounds (67), aldehydes (47), phenols (38), phenol ethers (30), acids (28), phenol esters (26), ethers (24), haloalkanes (5), and organosulfur compounds (4). These VOCs may be closely related to aroma accumulation in *R. fortunei*, among which esters and terpenoids were the two most abundant VOC categories in the petals. In the two treatment groups (BR and MeJA), the content of most VOCs was higher than that in the CK group (Figure 3C,D). Among them, compared with the CK group, 265 VOCs were upregulated and 70 were downregulated in the BR group, while 248 VOCs were upregulated and 181 were downregulated in the MeJA group.

### 3.3. Functional Enrichment Analysis of DAMs in R. fortunei

**Figure 4:**
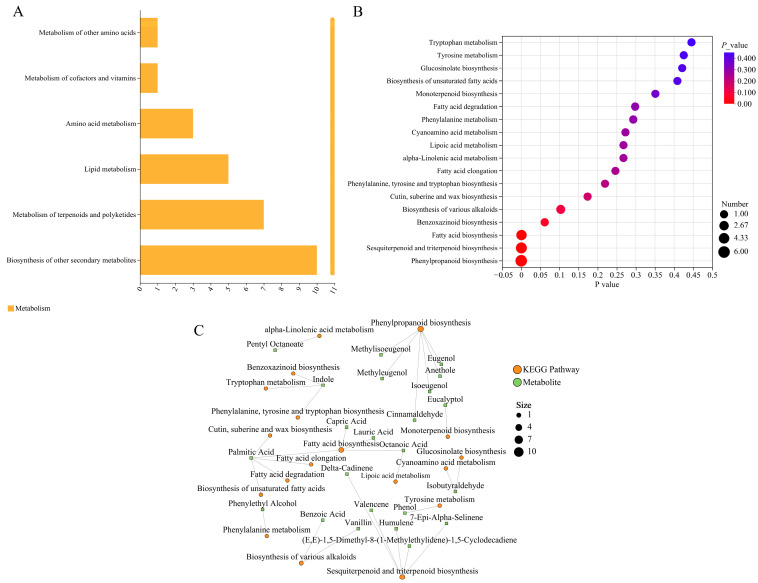
Functional enrichment analysis of DAMs. (**A**) KEGG pathway diagram. The x-axis represents the number of metabolites annotated to the pathway, and the y-axis represents the secondary categories of KEGG metabolic pathways. (**B**) KEGG bubble plot. The x-axis represents the enrichment significance *p*-value; the smaller the *p*-value, the greater the statistical significance, and a *p*-value < 0.05 is generally considered a significant enrichment term for the function. The y-axis represents KEGG pathways. The size of the bubble indicates the number of compounds enriched in the metabolite set within the pathway. (**C**) Correlation network diagram of KEGG pathways and metabolites. Orange represents KEGG pathways, green represents metabolites, and the lines represent direct associations between KEGG pathways and metabolites.

KEGG pathways can be divided into seven major categories, namely metabolism, genetic information processing, environmental information processing, cellular processes, organismal systems, human diseases, and drug development. Enrichment analysis showed that, except for “biosynthesis of other secondary metabolites”, more DAMs were enriched in the metabolism of terpenoids and polyketides and the biosynthesis of secondary metabolites (Figure 4A), while the KEGG bubble plot analysis indicated that more DAMs were enriched in phenylpropanoid biosynthesis, sesquiterpenoid and triterpenoid biosynthesis, and fatty acid biosynthesis (Figure 4B). Additionally, the hypergeometric test was used to analyze the significant pathway enrichment of DAMs in the metabolite set, with FDR < 0.05 set as the threshold for significant enrichment. The results (Figure 4C) showed that more DAMs were associated with phenylpropanoid biosynthesis (map00940), terpenoid backbone biosynthesis (map00900), and fatty acid biosynthesis (map00061), with six, five, and four associated DAMs, respectively.

### 3.4. VOCs Closely Related to Aroma Formation in R. fortunei

**Figure 5:**
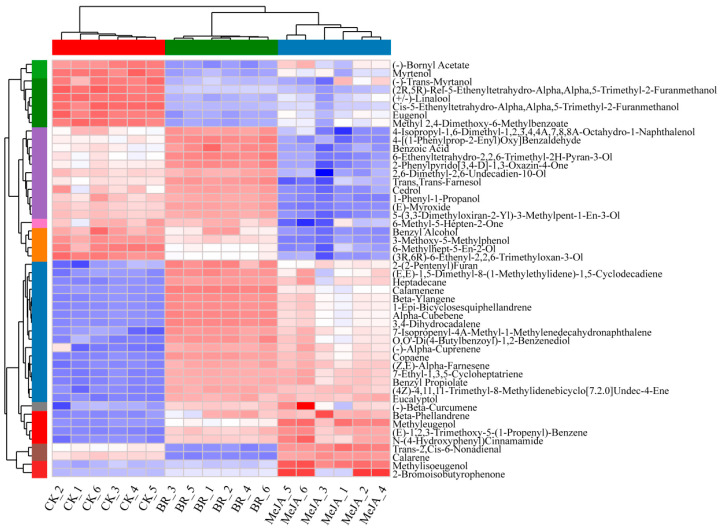
Comparative heatmap of 50 DAMs in three groups of *R. fortunei* samples. Red indicates relatively high metabolite abundance, and blue indicates relatively low abundance; the x-axis represents sample names, and the y-axis represents different DAMs; the color strip on the left side of the figure covers colors including green, purple, orange, blue, red and brown, where each distinct color corresponds to a specific sample group; the row and column hierarchical clustering trees show the similarity clustering relationships between metabolites and between samples, respectively.

As shown in the cluster heatmap of DAMs, samples in each group clustered into a single category independently and exhibited unique metabolite characteristics, indicating that the hormone treatments significantly altered the metabolite profiles of *R. fortunei* petals. Notably, among the 22 significantly upregulated DAMs, 12 were classified as terpenoids (Figure 5 and Table S4), specifically including (E,E)-1,5-dimethyl-8-(1-methylethylidene)-1,5-cyclodecadiene, calamenene, beta-ylangene, 1-epi-bicyclosesquiphellandrene, 3,4-dihydrocadalene, 7-isopropenyl-4A-methyl-1-methylenedecahydronaphthalene, (−)-alpha-cuprenene, copaene, (Z,E)-alpha-farnesene, (4Z)-4,11,11-trimethyl-8-methylidenebicyclo [7.2.0] undec-4-ene, (−)-beta-curcumene, and beta-phellandrene. It is worth noting that several additional metabolites also exhibited a distinct and consistent accumulation pattern. These included (−)-bornyl acetate, myrtenol, (−)-trans-myrtanol, (2R,5R)-rel-5-ethenyltetrahydro-α,α,5-trimethyl-2-furanmethanol, (+/−)-linalool, cis-5-ethenyltetrahydro-α,α,5-trimethyl-2-furanmethanol, eugenol, and methyl 2,4-dimethoxy-6-methylbenzoate, among others. This result provides an important basis for the further analysis of the metabolic regulation mechanisms mediated by different hormones and the screening of key aroma-related metabolites.

### 3.5. Overview of Transcriptome Sequencing

**Figure 6:**
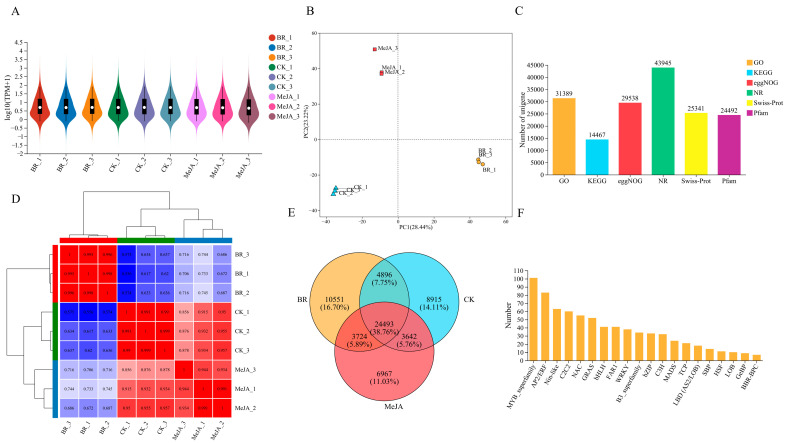
Overview of transcriptome sequencing. (**A**) Violin plot of transcriptome sequencing. The x-axis represents all biological replicates of different treatment groups; the outlines on both sides represent the distribution density of gene expression levels; the middle line of the boxplot represents the median; and the boxplot represents the interquartile range. (**B**) PCA plot. The x-axis represents the total variation of the first principal component (PC1), the y-axis represents the total variation of the second principal component (PC2), and points of different colors represent different sample groups. (**C**) Number of unigenes under different functional categories. The x-axis represents six functional annotation categories: GO, KEGG, eggNOG, NR, Swiss-Prot, and Pfam; the y-axis represents the corresponding number of unigenes. (**D**) Sample correlation heatmap. Both the x-axis and y-axis represent all biological replicates of different treatment groups, the color strip above the heatmap corresponds to different treatment groups, where each distinct color represents a specific group, displaying the gene expression patterns in different samples via a color gradient (blue → red indicates correlation from low to high). The hierarchical clustering trees on both sides represent the expression similarity of samples (closer clustering indicates more similar expression patterns). (**E**) Venn diagram of three sample groups. The three circles represent the gene sets of the BR, MeJA, and CK treatment groups, respectively; the numbers in each region correspond to the number of unique genes in each group and the number of common genes between groups. (**F**) Statistics of TFs. The x-axis represents the names of TFs, and the y-axis represents the number of TFs.

Three groups of *R. fortunei* petal samples (CK, BR, MeJA) after different hormone treatments were selected, with three biological replicates per group. RNA-seq analysis was performed using Illumina HiSeq high-throughput sequencing technology, yielding a total of 64.02 Gb of raw data and 62.71 Gb of clean data after filtering (Table S3). The violin plot results showed that replicate samples in the same treatment group had highly consistent box positions and density distribution shapes, indicating good intra-group experimental reproducibility, consistent gene expression patterns, and the high reliability of the experimental results (Figure 6A). Principal component analysis (PCA) further confirmed the good intra-group sample reproducibility and significant inter-group differences, verifying the reliability of the sequencing results (Figure 6B). The number of annotated unigenes in different functional databases was counted as follows (Figure 6C): 31,389 in the GO database, 14,467 in the KEGG database, 29,538 in the EggNOG database, 43,945 in the NR database, 25,341 in the Swiss-Prot database, and 24,492 in the Pfam database. The correlation heatmap analysis results (Figure 6D) showed high gene expression correlation between samples in the same group. In the clustering tree, samples from the same treatment group were first clustered into one branch, confirming the highly consistent gene expression patterns among intra-group replicate samples and good experimental reproducibility, while samples from different groups clustered at a higher level, indicating that the hormone treatments significantly altered the petal gene expression patterns and caused clear inter-group transcriptome differences. A Venn diagram was used to analyze the overlap and uniqueness of gene sets among the BR, MeJA, and CK groups (Figure 6E). The number of unique unigenes in the BR, MeJA, and CK groups was 10,551 (accounting for 16.70%), 8915 (accounting for 11.03%), and 6967 (accounting for 14.11%), respectively, and the number of common unigenes among the three groups was 24,493 (accounting for 38.76%). These common genes were speculated to be constitutively expressed genes or core regulatory genes that functioned under all three treatment conditions. In addition, the top 20 annotated transcription factor (TF) families were counted (Figure 6F), with the families ranked in descending order in terms of counts as follows: MYB, AP2/ERF, Nin-like, C2C2, NAC, GRAS, bHLH, FAR1, WRKY, B3, bZIP, C3H, MADS, TCP, LBD, SBP, HSF, LOB, GeBP, and BBR-BPC.

### 3.6. Differential Gene Analysis

**Figure 7:**
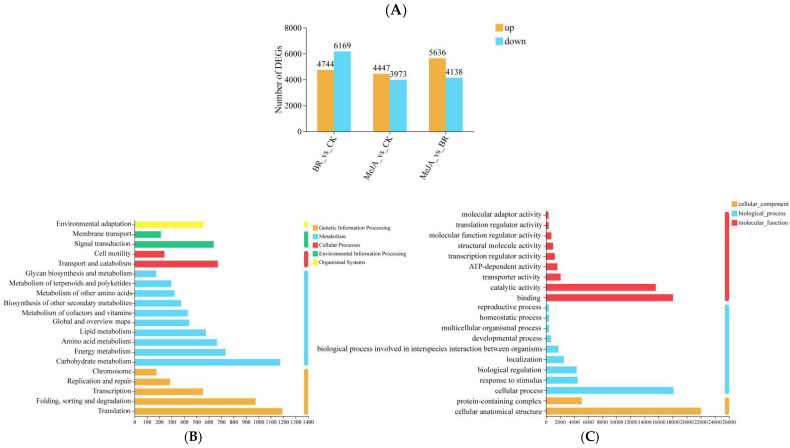
Differential gene analysis. (**A**) Statistics of DEGs among different groups. The x-axis represents the three different comparison groups, and the y-axis represents the number of DEGs; orange bars represent significantly upregulated genes, and blue bars represent significantly downregulated genes. (**B**) KEGG enrichment analysis. The x-axis represents the number of unigenes annotated to corresponding pathways, and the y-axis represents KEGG pathway names; the color legend on the right represents major KEGG functional categories. (**C**) GO enrichment analysis. The x-axis represents the number of unigenes annotated to corresponding terms, and the y-axis represents GO term names; the colors on the right represent major GO functional categories.

In the three comparison groups (BR vs. CK, MeJA vs. CK, and MeJA vs. BR), a large number of DEGs were detected, with significant differences in their upregulation/downregulation patterns (more downregulated genes in BR vs. CK and more upregulated genes in MeJA vs. BR) among the groups, suggesting that BRs and MeJA exhibit specificity in regulating gene expression in *R. fortunei* (Figure 7A). To reveal the biological functions of DEGs after BR and MeJA treatments, KEGG and GO functional enrichment analyses were performed on these DEGs, respectively (Figure 7B,C). The KEGG enrichment results showed that most DEGs were concentrated in metabolic pathways, which were further divided into 10 subcategories: carbohydrate metabolism, energy metabolism, amino acid metabolism, lipid metabolism, global and overview maps, metabolism of cofactors and vitamins, biosynthesis of other secondary metabolites, metabolism of other amino acids, metabolism of terpenoids and polyketides, and glycan biosynthesis and metabolism. These findings suggest that these metabolic pathways may be involved in the accumulation of unique aroma substances in *R. fortunei*. GO annotation was employed to classify DEGs into three major categories: biological process (BP), cellular component (CC), and molecular function (MF). Notably, the term with the largest number of annotated genes in the BP category was “cellular process” (18,129 genes in total), and the term with the largest number of annotated genes in the CC category was “cellular anatomical structure” (22,022 genes in total)—these genes may play important roles in the formation of floral aroma compounds in *R. fortunei*.

### 3.7. qRT-PCR Validation

**Figure 8:**
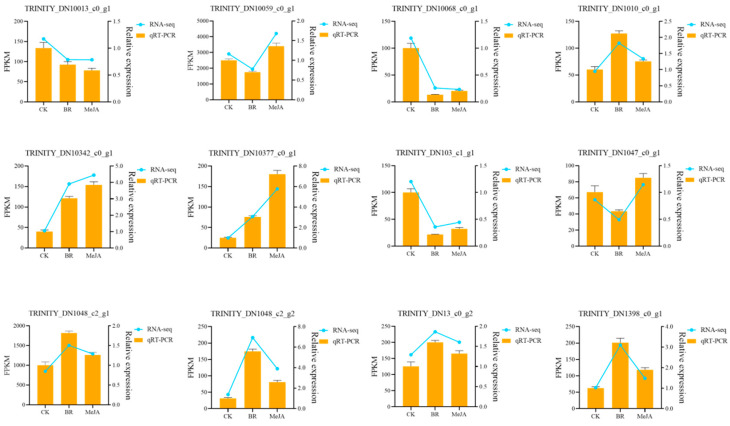
qRT-PCR validation. The x-axis represents different samples; the left y-axis represents FPKM values; the right y-axis represents qRT-PCR relative expression levels; orange bars represent the relative expression levels detected by qRT-PCR; the blue line represents the expression levels measured by FPKM in RNA-seq.

To verify the accuracy and reliability of the transcriptome analysis results obtained in this study, 12 DEGs were selected for the detection of their expression levels via qRT-PCR. The results showed that the qRT-PCR results for these 12 genes were largely consistent with those from transcriptome sequencing, indicating that the transcriptome data in this study were reliable (Figure 8).

### 3.8. Integrated Analysis of Transcriptome and Metabolome

To further explore the key DEGs and DAMs associated with aroma in *R. fortunei* after BR and MeJA treatments, Pearson’s correlation analysis was performed between DAMs and DEGs in each of the two comparison groups (MeJA vs. CK and BR vs. CK) (Table S5). Finally, 17 common DEGs were identified, namely TRINITY_DN1061_c2_g1, TRINITY_DN15383_c0_g1, TRINITY_DN38502_c0_g2, TRINITY_DN2506_c0_g1, TRINITY_DN473_c0_g1, TRINITY_DN14618_c0_g1, TRINITY_DN49_c0_g1, TRINITY_DN31_c0_g1, TRINITY_DN2686_c0_g1, TRINITY_DN3755_c2_g1, TRINITY_DN12516_c0_g1, TRINITY_DN2373_c0_g1, TRINITY_DN2065_c0_g1, TRINITY_DN2429_c0_g1, TRINITY_DN78_c0_g1, TRINITY_DN2432_c0_g1, and TRINITY_DN25546_c0_g2. After annotating these 17 genes using the Swiss-Prot database, it was found that only four of these DEGs were annotated successfully, among which two DEGs (TRINITY_DN15383_c0_g1, TRINITY_DN14618_c0_g1) were annotated as *CYP92C6*.

### 3.9. Cloning and Subcellular Localization of RfCYP92C6

**Figure 9:**
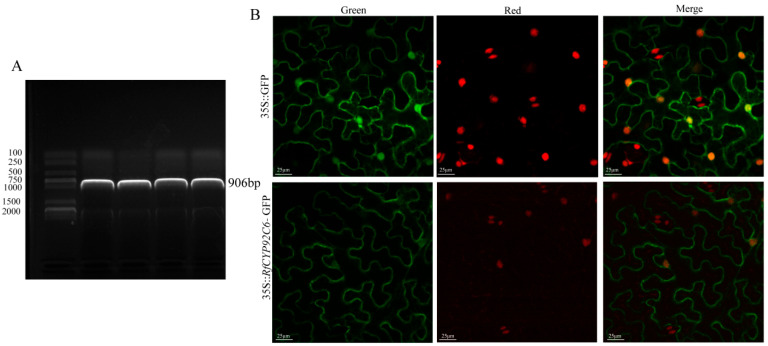
Cloning and subcellular localization of *RfCYP92C6*. (**A**) Cloning of *RfCYP92C6*: electrophoretic analysis of PCR amplification products of the target gene. The left side shows the DNA marker (DNA molecular weight standard); the labeled bands represent DNA fragments of different lengths, which were used to determine the sizes of sample bands. In the sample lane on the right, a clear band appears, whose position corresponds to a molecular weight of 906 bp. (**B**) Subcellular localization of *RfCYP92C6*: the upper row shows the 35S::GFP control (“Green” represents GFP fluorescence, “Red” represents chloroplast autofluorescence, and “Merge” represents the merged image); the lower row shows the localization results of the 35S::*RfCYP92C6*-GFP fusion protein, displaying the subcellular distribution characteristics of the target protein.

*RfCYP92C6* was successfully cloned from *R. fortunei* (Figure 9A). For the subcellular localization assay, the empty vector pCAMBIA1300-GFP served as the negative control, with no specific green fluorescent signal detected. In contrast, for the overexpression vector pCAMBIA1300-*RfCYP92C6*-GFP, a specific green fluorescent signal was observed at the plasma membrane in *N. benthamiana* leaf cells (Figure 9B). This observation was consistent with the predicted subcellular localization.

### 3.10. Analysis of Spatiotemporal Expression Pattern of RfCYP92C6

**Figure 10:**
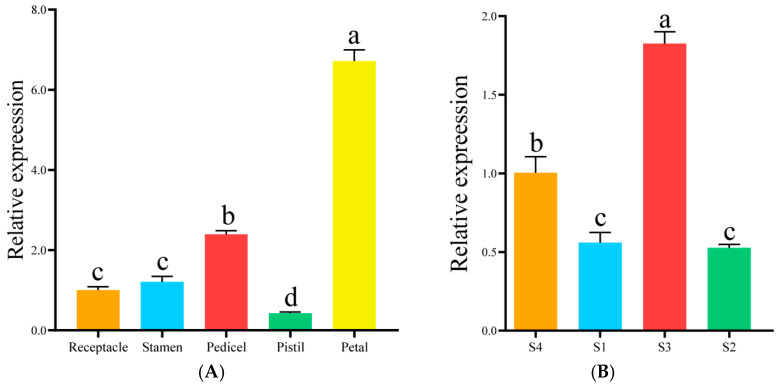
Analysis of spatiotemporal expression pattern of *RfCYP92C6*. (**A**) Expression pattern of *RfCYP92C6* in different organs. The x-axis represents different organs of *R. fortunei*, and the y-axis represents relative expression. (**B**) Expression pattern of *RfCYP92C6* in different developmental stages. The x-axis represents different developmental stages of *R. fortunei*, and the y-axis represents relative expression. Different letters (a, b, c, d) at the tops of columns indicate significant differences among groups after multiple comparisons, while the same letters indicate no significant differences among groups.

The relative expression level of *RfCYP92C6* in *R. fortunei* petals was significantly higher than that in the pedicels, stamens, receptacles, and pistils, with the lowest level detected in the pistils (Figure 10A). Meanwhile, the relative expression level of this gene in the S3 stage was significantly higher than that in the S4 stage, and its expression levels were relatively low in the S1 and S2 stages (Figure 10B).

### 3.11. Functional Identification of RfCYP92C6 in N. benthamiana

**Figure 11:**
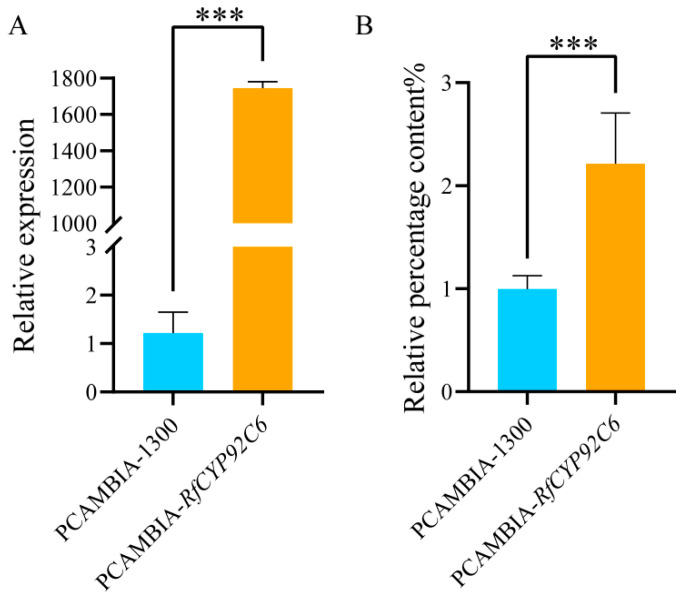
Functional identification of *RfCYP92C6* in *N. benthamiana*. (**A**) Detection of the relative expression level of *RfCYP92C6* in *N. benthamiana* leaves via qRT-PCR. (**B**) Relative content of terpenoids in *N. benthamiana* leaves after transient overexpression of *RfCYP92C6*, with PCAMBIA-1300 as the empty vector control and PCAMBIA-*RfCYP92C6* as the *RfCYP92C6* overexpression group; *t*-test results showed *p* < 0.001 (significance level), where “***” indicates extremely significant differences at the 0.001 level.

To further explore the function of *RfCYP92C6*, the transient overexpression of this gene was performed in *N. benthamiana* leaves. The qRT-PCR results showed that the expression level of *RfCYP92C6* in the overexpression group was significantly higher than that in the control group with the empty vector pCAMBIA1300 (Figure 11A). Moreover, the gas chromatography–mass spectrometry (GC-MS) detection results indicated that, compared with the empty vector control group, the content of terpenoids in the overexpression leaves increased 2.2-fold (Figure 11B). These results demonstrate that *RfCYP92C6* plays an important role in the synthesis of terpenoids—the key components of the floral aroma in *R. fortunei*.

## 4. Discussion

The floral aroma consists of various VOCs, which play a crucial role in plant growth and development and are released into the surrounding air from plant floral organs [28,33]. With the increasing attention being paid to ornamental plants, the demand for improved aromas is also increasing. The formation of aromas in ornamental plants is a complex process. Exogenous plant hormones can promote plant growth and development, and this study focused on analyzing the aroma changes in *R. fortunei* following different hormone treatments. More specifically, we performed a comparative analysis on *R. fortunei* treated with BRs and MeJA. By integrating metabolomic and transcriptomic data, we found that genes such as *RfCYP92C6* were specifically expressed in different treatment groups. Notably, in the petal samples of *R. fortunei* treated with the BR and MeJA, significant changes were observed in the content of compounds such as methyleugenol, calamenene, and copaene, suggesting that these compounds may play a key role in the aroma formation of *R. fortunei*.

In recent years, numerous studies have identified key regulatory genes involved in terpenoid biosynthesis in ornamental plants through integrated metabolomic and transcriptomic analyses [34,35,36]. In this study, terpenoid biosynthesis was identified as the key metabolic pathway contributing to aroma changes in *R. fortunei.* By comparing the BR and MeJA treatment groups, we identified a total of 22 types of VOCs. Furthermore, by integrating volatile metabolome analysis with RNA-seq data, we detected a strong correlation between metabolite content and gene expression, and 17 DEGs were finally identified. These 17 genes were annotated using the Swiss-Prot database; only four of these DEGs were successfully annotated, among which two DEGs were annotated as *RfCYP92C6*. Therefore, we selected the candidate gene *RfCYP92C6* for preliminary functional validation.

Cytochrome P450 enzymes (CYPs) are widely distributed in various plants, and CYP genes are involved in almost all molecular processes in plants. Over the past two decades, the scope of CYP gene identification has expanded rapidly, and studies have identified more than 40,000 *CYP* genes and 819 CYP families [37,38]. However, there is still a lack of systematic and comprehensive reviews on plant CYP family genes. *N. benthamiana*, as a commonly used model plant, plays an important role in plant gene regulation. In this study, the overexpression of *RfCYP92C6* promoted the production of terpenoid aroma substances in *N. benthamiana*, indicating that this gene may play an important role in heterologous aroma production in *N. benthamiana*, and we hypothesize that it functions as a positive regulatory factor in floral aroma formation in *R. fortunei*. However, the specific mechanisms by which *RfCYP92C6* participates in floral aroma regulation at the protein level still require further experimental verification.

The activity of SOD and CAT can indirectly reflect the physiological vitality of *R. fortunei*. In this study, after treatment with the two hormones (BR and MeJA), the activity of SOD and CAT in *R. fortunei* was increased, while the content of MDA was significantly decreased. As key enzymes in the plant antioxidant defense system, SOD and CAT exhibited increased activity, which indicates that the ability of *R. fortunei* to scavenge ROS was enhanced, thereby effectively alleviating cell damage caused by oxidative stress. Meanwhile, MDA is a hallmark product of membrane lipid peroxidation, and a reduction in its content suggests that cell membrane integrity is better preserved. The above results indicate that treatment with the two hormones can enhance the oxidative stress resistance of *R. fortunei* by activating its antioxidant enzyme system. This may be one of the most important physiological mechanisms by which these two hormones regulate aroma accumulation in *R. fortunei*, and this finding provides a theoretical basis for the further exploration of the regulatory role and application value of BRs and MeJA in *Rhododendron* spp.

TFs can regulate the expression of many genes related to floral aromas and have received extensive attention [39,40,41]. In this study, we identified transcription factors from families such as MYB, AP2/ERF, Nin-like, and C2C2, and numerous studies have confirmed that these TFs play regulatory roles in the production of volatile compounds in flowers. For example, in *Jasminum sambac*, *LHY* was identified as a core TF. Through DNA-seq, a dual-luciferase reporter gene assay, and a yeast one-hybrid assay, it was verified that *JsLHY* is involved in the aroma formation of jasmine [31]. In *Lilium* ‘*Siberia*’, through the transient overexpression of *bHLH22* and *bHLH63* in petals and virus-induced gene silencing (VIGS) assays, we confirmed that these TFs effectively regulate floral aroma formation. Nonetheless, the regulatory mechanisms of related TFs in the floral aroma formation of *R. fortunei* remain to be further explored through in-depth research in the future.

The results of this study indicate that BRs and MeJA can enhance the aroma of *R. fortunei* by strengthening the antioxidant defense mechanism and regulating the accumulation of primary and secondary metabolites and the expression of related genes. These findings lay a foundation for the elucidation of the regulatory mechanisms underlying the biosynthesis of floral aroma compounds in *R. fortunei* and provide a theoretical basis for improvements in the aromas of common *Rhododendron* cultivars.

## 5. Conclusions

This study integrated physiological and biochemical assays, transcriptomics, and volatile metabolomics to clarify the molecular mechanism underlying BR-/MeJA-mediated aroma biosynthesis in *R. fortunei* petals. This study identified 1268 VOCs (including 198 terpenoids—the key aroma components) and screened aroma-related candidate genes, and preliminary functional analysis revealed that *RfCYP92C6* (highly expressed in S3-stage petals) mediates the floral aroma by promoting terpenoid biosynthesis—its transient overexpression in *N. benthamiana* increased terpenoids 2.2-fold. However, the specific regulatory mechanisms of this gene and other genes in the processes of aroma biosynthesis require further exploration and verification through subsequent experiments. Taken together, this study provides an important theoretical basis for improvements in the aroma traits of *R. fortunei* through genetic engineering or marker-assisted breeding in the future, with the aim of further enhancing the ornamental and economic value of *Rhododendron* spp. and other horticultural plants.

## Data Availability

The original contributions presented in this study are included in the article/Supplementary Materials. Further inquiries can be directed to the corresponding authors.

## Supplementary Materials

The following supporting information can be downloaded at: https://www.mdpi.com/article/10.3390/cimb47121054/s1.
