# Supplementary material for: Multi-Omics Provides New Insights into the Aroma Regulation of Rhododendron fortunei Lindl Treated with Methyl Jasmonate and Brassinosteroids
Source: Curr Issues Mol Biol. 2025 Dec 16;47(12):1054. doi: 10.3390/cimb47121054 (PMC12731677; doi:10.3390/cimb47121054)
Supplement: Supplementary file 1 [file cimb-47-01054-s001.zip › Table S1. All primers in this study.pdf]

	Gene ID	Forward primer (5’-3’)	Reverse primer (5’-3’)
qRT-PCR	*TRINITY_DN10013_c0_g1_1*	TCGGTAAGGCCTCCGGAT	ATCCAGCGTGTGACGTGG
	TRINITY_DN10059_c0_g1_1	CGTCGGGAGAGGGTACGA	CACAAGGTTGATGCCATCGAC
	TRINITY_DN10068_c0_g1_1	GGGTTTGGGTGTGCTGGT	GGACGAACACCACAAGAGC
	TRINITY_DN1010_c0_g1_1	CACCACCAGAAGCCTCGG	GGCAGATGAGTGGCTCCC
	TRINITY_DN10342_c0_g1_1	CACCGTCTCCCTCCCTCA	TGGTGTTGTGGGGGAGGA
	TRINITY_DN10377_c0_g1_1	GCGAGCTGATGGGGTTGT	CGCTCAGAGCAACCACCA
	TRINITY_DN103_c1_g1_1	CGGTCCACCAATCCGCTT	GGTCGAGAACGGTGGTGG
	TRINITY_DN1047_c0_g1_1	AGGGCCAGAGATGCTCCA	GCTCGAGCCAGATCCCAC
	TRINITY_DN1048_c2_g1_1	CACCTGCACCACTCCTCG	GGTCGCGGTCTGCTTCTT
	TRINITY_DN1048_c2_g2_1	AAGGACGTTGAGGTGGCG	ATGTCTATCAGCCGCGCC
	TRINITY_DN13_c0_g2_1	GCTCGTGATTGCAGCAGC	CAGGGACACGGACCAACC
	TRINITY_DN1398_c0_g1_1	TGCACTCGAAGCTGGTGG	GTGCACGTCAAGGCAAGC
	EF1α	AGACCACCAAGTACTACTGCAC	CCACCAATCTTGTACACATCC
Cloning and DNA testing	*RfCYP92C6*	ATGGAAACTCCGTCGTGGACC	CTGGGTGAAAGCCTTGACCC
